# FM19G11-Loaded Gold Nanoparticles Enhance the Proliferation and Self-Renewal of Ependymal Stem Progenitor Cells Derived from ALS Mice

**DOI:** 10.3390/cells8030279

**Published:** 2019-03-23

**Authors:** Stefania Marcuzzo, Davide Isaia, Silvia Bonanno, Claudia Malacarne, Paola Cavalcante, Antonella Zacheo, Valentino Laquintana, Nunzio Denora, Barbara Sanavio, Elisa Salvati, Patrizia Andreozzi, Francesco Stellacci, Silke Krol, Maravillas Mellado-López, Renato Mantegazza, Victoria Moreno-Manzano, Pia Bernasconi

**Affiliations:** 1 Neurology IV—Neuroimmunology and Neuromuscular Diseases Unit, Fondazione IRCCS Istituto Neurologico “Carlo Besta”, 20133 Milan, Italy; silvia.bonanno@istituto-besta.it (S.B.); malacarne.c17@gmail.com (C.M.); paola.cavalcante@istituto-besta.it (P.C.); renato.mantegazza@istituto-besta.it (R.M.); 2 Institut de Génétique et de Biologie Moléculaire et Cellulaire (IGBMC), Department of Development and Stem Cells, CNRS UMR7104, INSERM U964, Université de Strasbourg, 67404 Illkirch CU Strasbourg, France; isaiad@igbmc.fr; 3 Laboratory for Translational Nanotechnology, IRCCS Istituto Tumori “Giovanni Paolo II”, 70124 Bari, Italy; zacheo@yahoo.com (A.Z.); silke.krol@aol.com (S.K.); 4 Department of Pharmacy—Pharmaceutical Sciences, University of Bari “Aldo Moro”, 70124 Bari, Italy; valentino.laquintana@uniba.it (V.L.); nunzio.denora@uniba.it (N.D.); 5 Laboratory for Nanomedicine, Fondazione IRCCS Istituto Neurologico “Carlo Besta”, 20133 Milan, Italy; barbara.sanavio@gmail.com; 6 IFOM, the FIRC Institute for Molecular Oncology Foundation, IFOM-IEO Campus, 20139 Milan, Italy; eli.salvati@gmail.com; 7 CIC biomaGUNE, 20014 San Sebastian, Spain; pandreozzi@cicbiomagune.es; 8 Institute of Materials, Ecole Polytechnique Fédérale de Lausanne (EPFL), STI IMX SUNMIL, MXG 030 (Bâtiment MXG), CH-1015 Lausanne, Switzerland; francesco.stellacci@epfl.ch; 9 IRCCS Azienda Ospedaliera Specializzata in Gastroenterologia “Saverio de Bellis”, 70013 Castellana Grotte, Italy; 10 Neuronal and Tissue Regeneration Laboratory, Prince Felipe Research Institute (CIPF), 46512 Valencia, Spain; mmellado@cipf.es (M.M.-L.); vmorenom@cipf.es (V.M.-M.)

**Keywords:** amyotrophic lateral sclerosis, ependymal stem progenitor cells, FM19G11, G93A-SOD1 mouse model, nanoparticles

## Abstract

Amyotrophic lateral sclerosis (ALS) is a progressive neurodegenerative disease affecting motor neurons. In ALS mice, neurodegeneration is associated with the proliferative restorative attempts of ependymal stem progenitor cells (epSPCs) that normally lie in a quiescent in the spinal cord. Thus, modulation of the proliferation of epSPCs may represent a potential strategy to counteract neurodegeneration. Recent studies demonstrated that FM19G11, a hypoxia-inducible factor modulator, induces epSPC self-renewal and proliferation. The aim of the study was to investigate whether FM19G11-loaded gold nanoparticles (NPs) can affect self-renewal and proliferation processes in epSPCs isolated from G93A-SOD1 mice at disease onset. We discovered elevated levels of SOX2, OCT4, AKT1, and AKT3, key genes associated with pluripotency, self-renewal, and proliferation, in G93A-SOD1 epSPCs at the transcriptional and protein levels after treatment with FM19G11-loaded NPs. We also observed an increase in the levels of the mitochondrial uncoupling protein (*UCP*) gene in treated cells. FM19G11-loaded NPs treatment also affected the expression of the cell cycle-related microRNA (miR)-19a, along with its target gene PTEN, in G93A-SOD1 epSPCs. Overall our findings establish the significant impact of FM19G11-loaded NPs on the cellular pathways involved in self-renewal and proliferation in G93A-SOD1 epSPCs, thus providing an impetus to the design of novel tailored approaches to delay ALS disease progression.

## 1. Introduction

Amyotrophic lateral sclerosis (ALS) is a progressive neurodegenerative disease that primarily affects motor neurons in the motor cortex (upper motor neurons), brain stem, and spinal cord (lower motor neurons). While most cases are sporadic in nature (sALS), 10% of cases take the hereditary familial forms (fALS), of which approximately 20% are caused by mutations in the superoxide dismutase 1 (SOD1) gene [1,2]. Both sALS and fALS patients present with a loss of upper and lower motor neurons, thereby resulting in similar pathologies [3].

The G93A-SOD1 transgenic mouse model of ALS overexpresses a mutant form of the human SOD1 gene [4,5] and displays detectable clinical signs and neuropathological features that mimic fALS, including severe hind limb paralysis and skeletal muscle atrophy [6,7,8]. In this ALS model mice, neurodegeneration promotes the proliferative restorative actions of ependymal stem progenitor cells (epSPCs) that are typically present in the spinal cord in a quiescent state [9,10,11]. epSPCs derived from the post-mortem spinal cord of ALS mice and human patients have the ability to differentiate into the three neural cell lineages in vitro [11,12]. Consequently, the modulation of the proliferative capacity of epSPCs may represent an effective strategy to arrest neurodegeneration and the progression of the disease.

Recent studies have demonstrated that FM19G11, a hypoxia-inducible factor modulator (HIF), boosts the self-renewal and proliferation of epSPCs [13]. Directed differentiation experiments demonstrated that FM19G11 also induced oligodendrocyte differentiation of epSPCs isolated from an adult rat model of spinal cord injury, which is normally blocked under hypoxia, possibly through the negative modulation of SRY-related HMG-box 2 (SOX2) and octamer-binding transcription factor 4 (OCT4) expression [13]. Furthermore, FM19G11 treatment modifies the mitochondrial uncoupling process, resulting in a transient reduction in ATP levels. This then triggers an adaptive cellular response, inducing a compensatory increase in glucose uptake via the activation of AMP-activated protein kinase (AMPK) [14,15,16] and protein kinase B (AKT) signaling pathways [17] to provide energy for subsequent ATP production [18,19]. FM19G11 treatment of epSPCs led to an overall increase in ATP levels and resulted in increased cell proliferation, leading to the investigation of FM19G11 treatment in a rat model of spinal cord injury. Interestingly, intrathecal infusion of FM19G11 induced functional locomotion recovery and reduced the number of glial fibrillary acidic protein (GFAP)-positive activated astrocytes at the site of injury [19]. However, the hydrophobic nature of FM19G11 represents a significant obstacle to clinical application in human patients.

In the present study, we investigated how treatment with FM19G11-loaded gold nanoparticles (NPs) affects the self-renewal and proliferation of epSPCs isolated from G93A-SOD1 mice and B6.SJL control mice at times representative of various stages of the disease (pre-symptomatic phase, disease onset, and late symptomatic phase). A comprehensive analysis, including NP kinetics, targeted histology, and gene/protein expression, demonstrated the significant impact of FM19G11-loaded NPs on the activation of cellular pathways involved in the self-renewal and proliferation of G93A-SOD1 epSPCs. We believe that this study will help to inform the design of novel tailored approaches to treat ALS and delay disease progression in human patients.

## 2. Materials and Methods

### 2.1. Animals

Transgenic G93A-SOD1 (B6SJL-Tg(SOD1*G93A)1Gur/J) and B6.SJL mice purchased from Charles River, Inc. (Wilmington, MA, USA) were maintained and bred at the animal house of the Carlo Besta Neurological Institute, in compliance with institutional guidelines and international regulations (EEC Council Directive 86/609), and project approvals were sought from the Italian Ministry of Health (reference number IMP-01-12; 183/2018-PR). G93A-SOD1 progeny were identified via real-time (RT)-PCR analysis using primers specific to the human SOD1 gene [7]. Male mice were used in all studies and sacrificed by exposure to carbon dioxide at different post-natal weeks: 8 (pre-symptomatic phase), 12 (disease onset), and 18 (late symptomatic phase). Ten animals per group for each time point were used. Cell lines were isolated from all animals and treated as described below; of these cell lines, three were used for cellular experiments, five for molecular analyses, and two for Western blot analyses.

### 2.2. Nanoparticle Preparation and Drug Loading

Gold NPs coated with 11-mercapto-1-undecanesulphonate (MUS) and 1-octanethiol (OT) (hereafter referred to as MUS:OT NPs) were prepared following the protocol of Verma et al. [20]. The ligand shell was loaded with FM19G11 (F8807, Sigma-Aldrich, St. Louis, MO, USA) according to the method described by Yang et al. [21]. A publication providing a detailed protocol and full characterization of FM19G11-loaded NPs is currently under preparation. In brief, for the preparation of 50 µM FM19G11-loaded NPs, 25 µL of MUS:OT NPs (dissolved in DMSO, 10 mg/mL) were mixed with 25 µL FM19G11 (working solution 0.5 mM in DMSO) or with 25 µL DMSO as NP control and vortexed for 30 secs. Subsequently, 200 µL of water were added dropwise vortexing so that the ever-increasing hydrophilic nature of the solvent drove more hydrophobic drug into the ligand shell. The mixture was then sterile-filtered through a 0.2-µm pore size membrane.

For the synthesis of fluorescently labeled NPs, 6-(((4,4-difluoro-5-(2-thienyl)-4-bora-3a,4a-diaza-s-indacene-3-yl)styryloxy)acetyl)-aminohexanoic acid, succinimidyl ester (BODIPY 630/650-X, SE) were purchased from Life Technologies and thiolated on the succinimidyl ester group to prepare BODIPY-SH [20]. Next, a stock solution was prepared by solving 3.7 mg of BODIPY-SH in 1.5 mL of a deionized water/dimethylformamide mixture (2:1 vol:vol). In a ligand exchange reaction, a proportion of ligands from the MUS:OT NPs become replaced by the thiolated fluorophore. Therefore, 10 mg of MUS:OT NPs (~10^−8^ moles for 4.5 nm particles) were dissolved in 0.75 mL of deionized water. Then, 75 μL (8 × 10^−7^ moles) of the BODIPY-SH stock solution were added to the NP solution and stirred for two days in a sealed and dark environment following the protocol described by Templeton et al. [22]. Finally, the samples were washed with acetone and pelleted by centrifugation (13,000 rpm, 5 min, 10 °C). The pellet consists of the fluorescently labeled NPs, while the free fluorophore remains in solution. The pellet was washed at least five times by resuspension and centrifugation.

### 2.3. Culture of epSPCs and FM19G11 Treatment

The epSPCs were isolated from whole spinal cords of 8-, 12-, and 18-week-old G93A-SOD1 and age-matched B6.SJL mice. After removal of the overlying meninges and blood vessels, spinal cords were cut into small pieces, dissociated with 0.05% collagenase I (Thermo Fisher Scientific, Foster City, CA, USA) for 15 min at 37 °C, and then processed to produce epSPC neurospheres as previously described [11]. On day 7, epSPC neurospheres were dissociated into single cells (cell passage 1, P1), counted by Trypan blue exclusion, and then seeded and cultured for another week. This was repeated until day 20 (P3) of in vitro culture to generate sufficient cell numbers for analyses and to ensure that cultures were devoid of non-neurosphere forming cells. Neurospheres were monitored periodically by optical microscopy (Eclipse TE 2000-S, Nikon, Tokyo, Japan). At P3, epSPCs from G93A-SOD1 and B6.SJL mice were cultured at a density of 9 × 10^5^ cells in proliferative medium at different growth conditions: (1) basal control conditions; (2) FM19G11 (Sigma) treatment (500 nM of FM19G11 and 500 nM DMSO, Sigma), and, separately, the corresponding amount of vehicle as control; and (3) FM19G11-loaded NP treatment (500 nM of FM19G11 and 0.01 mg/mL NPs) and, separately, the corresponding amount of vehicle as control. Cells were maintained in culture for 24 and 48 h. Then, epSPC neurospheres were dissociated into single cells and counted by Trypan blue exclusion.

### 2.4. Real-Time PCR Analyses of Gene Expression

Total RNA was extracted using TRIzol from 1 to 2 × 10^6^ epSPCs cultured under the various conditions. RNA quality was verified using a 2100 Nano Bioanalyzer (Agilent Technologies, Waldbron, Germany). Total RNA was retrotranscribed using the SuperScript Vilo cDNA Synthesis kit. cDNA (corresponding to 100 ng total RNA) was amplified by quantitative (q)RT PCR, in triplicate, using Universal PCR master mix and TaqMan Array Fast Plate assembled by Thermo Fisher Scientific with primer and probe sets for the *TERT*, *AKT1*, *AKT2*, *AKT3*, *UCP1*, *UCP2*, *PTEN*, *SOX2*, and *OCT4* genes and the 18S housekeeping gene on an Applied Biosystems PRIMS 7500 Fast Real-Time PCR System. mRNA expression levels were normalized against 18S, and the relative mRNA expression levels were calculated using the formula 2^−ΔCt^.

### 2.5. Real-Time PCR Analysis of miR-19a and miR-19b

Total RNA, previously examined for mRNA expression in epSPCs, was retrotranscribed to cDNA using TaqMan MicroRNA Reverse Transcription Kits with primers specific for miR-19a, miR-19b, and U6. U6 was stably expressed in epSPCs from G93A-SOD1 and B6.SJL mice at different conditions and used as a control. cDNA aliquots corresponding to 15 ng total RNA were amplified by qRT PCR in triplicate, with Universal PCR master mix and specific pre-designed TaqMan MicroRNA assays (Thermo Fisher Scientific). miRNA levels were normalized to U6 and expressed using the formula 2^−ΔCt^.

### 2.6. Western Blot Analyses

epSPCs were collected and protein extracted using 2% SDS-TrisCl lysis buffer. Here, 20 μg of protein were loaded per lane in 10% SDS-polyacrylamide gels and resolved by standard SDS-PAGE. Proteins were electrophoretically transferred onto polyvinylidene difluoride (PVDF) membranes, which were blocked with 5% non-fat dry milk in phosphate-buffered saline supplemented with Tween 20 (PBST) for 60 min and incubated overnight with specific antibodies against SOX2, OCT4, PTEN (Abcam, Cambridge, UK), and p-AKT (Cell Signaling, Danvers, MA, USA) at a 1:500 dilution. β-actin at a dilution of 1:5000 (Sigma) was used as a loading control. Subsequently, membranes were incubated with anti-mouse, anti-rabbit, or anti-goat horseradish peroxidase-conjugated secondary antibodies (1:5000) (Sigma). Blots were visualized with the ECL (Amersham, UK) detection system.

### 2.7. Statistical Analysis

Data from epSPC quantification, qRT PCR analyses, and densitometry analysis of Western blot bands were analyzed and charted using the GraphPad Prism7 software (GraphPad Inc., La Jolla, CA, USA). Significant differences were inferred at *p* < 0.05 as given by comparison among more than two groups by one-way analysis of variance (ANOVA) followed by Bonferroni post-hoc test.

## 3. Results

### 3.1. FM19G11-Loaded NP Treatment Boosts Percentage of epSPCs

**Figure 1:**
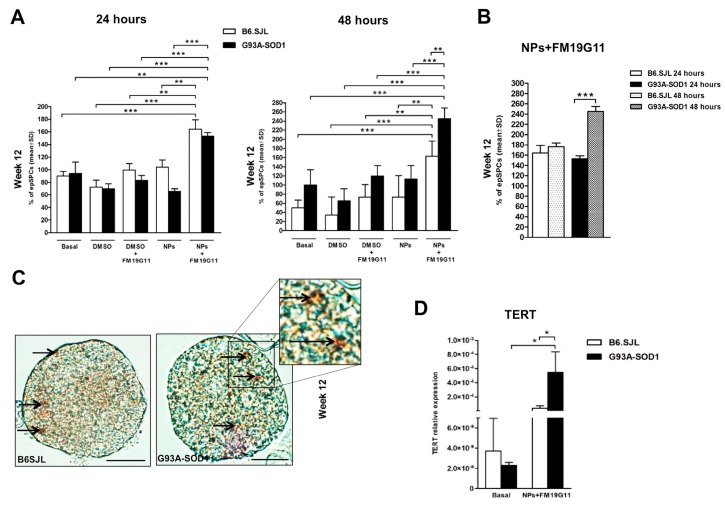
Effect of FM19G11 treatment on the percentage of G93A-SOD1 and control B6.SJL ependymal stem progenitor cells (epSPCs) and telomerase reverse transcriptase (*TERT*) expression. (**A**) Percentage of 12-week B6.SJL (white bars) and G93A-SOD1 (black bars) epSPCs after 24 and 48 h in the following growth conditions: (1) growth medium alone as the basal control condition; (2) FM19G11 treatment and, separately, DMSO alone as vehicle control; and (3) FM19G11-loaded nanoparticle (NP) treatment and, separately, non-loaded NPs as control. (**B**) Percentage of B6.SJL and G93A-SOD1 epSPCs following 24 (white and black bars) and 48 (white and black dot patterns) hours of treatment with FM19G11-loaded NPs. Data presented as mean ± SD of epSPC percentage (*n* = 10 different cell cultures from 10 animals per group). (**C**) Bright light microscopy images of B6.SJL and G93A-SOD1 epSPC neurospheres treated with BODYPY-labelled NPs. Arrows indicate NPs within cells, with higher magnification displayed in the inset. Scale bars: 20 μm. (**D**) qRT PCR expression analysis of the *TERT* gene in B6.SJL (white bars) and G93A-SOD1 (black bars) epSPCs after 48 h of treatment with FM19G11-loaded NPs compared with basal conditions. Expression levels are reported as mean ± SD of 2^−ΔCT^ values normalized against the endogenous control 18S (*n* = 5 different primary cell cultures from five animals per group). * *p* < 0.05, ** *p* < 0.01, *** *p* < 0.001 by ANOVA followed by Bonferroni post-hoc test.

To identify the optimal FM19G11 treatment conditions, we cultured epSPCs isolated from G93A-SOD1 and B6.SJL mice at times representative of the pre-symptomatic phase (8 weeks), disease onset (12 weeks), and late symptomatic phase (18 weeks) for 24 and 48 h under the following conditions: (1) growth medium alone as the basal control condition; (2) FM19G11 treatment and, separately, DMSO alone as vehicle control; and (3) FM19G11-loaded NP treatment and, separately, non-loaded NPs as control (Figure 1A,B and Figure S1). We confirmed the uptake of NPs by B6.SJL and G93A-SOD1 neurospheres via treatment with BODIPY-labelled NPs and subsequent visualization by fluorescence microscopy (Figure 1C).

We discovered a significant increase in the percentage of epSPCs derived from 12-week B6.SJL and G93A-SOD1 mice after 24 and 48 h of FM19G11-loaded NP treatment compared to other culture setups (Figure 1A). For 8-week G93A-SOD1 and B6.SJL epSPCs, we also observed an increased percentage of cells after FM19G11-loaded NP treatment, but we failed to find significant differences in all comparisons (Figure S1). When comparing 24 h treatment to 48 h treatment with FM19G11-loaded NPs, we observed an increase in the percentage of 8-week (Figure S1) and 12-week (Figure 1B), but not with 18-week epSPCs.

Given these findings, we chose the 48 h treatment for further analysis. To further explore the effect of FM19G11-loaded NPs on epSPC proliferation, we analyzed the expression of telomerase reverse transcriptase (TERT) component through qRT PCR. We observed a significant increase in TERT mRNA levels in FM19G11-loaded NPs treated 12-week G93A-SOD1 epSPCs when compared to FM19G11-loaded NP treated B6.SJL epSPCs and to G93A-SOD1 epSPCs at basal levels (untreated) (Figure 1D). We also discovered a significant increase in TERT mRNA levels in FM19G11-loaded NP-treated 8-week B6.SJL and G93A-SOD1 epSPCs compared to untreated epSPC controls (Figure S1; *p* < 0.05). At 18 weeks, we failed to observe differences in TERT mRNA levels between treated and untreated cells in the two groups of animals (Figure S1).

### 3.2. FM19G11-Loaded NPs Increase Expression of the SOX2 and OCT4 Pluripotency Markers in G93A-SOD1 epSPCs

**Figure 2:**
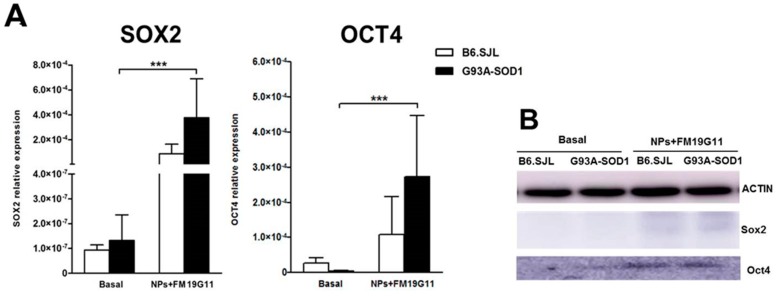
Analyses of SOX2 and OCT4 pluripotency marker expression. (**A**) qRT PCR expression analysis of *SOX2* and *OCT4* genes in 12-week B6.SJL (white bars) and G93A-SOD1 (black bars) epSPCs after 48 h of FM19G11-loaded NP treatment and under basal conditions. Expression levels reported as mean ± SD of 2^−ΔCT^ values normalized against the endogenous control 18S (*n* = 5 different primary cell cultures from five animals per group). (**B**) Representative Western blot analysis of SOX2, OCT4, and β-actin (*n* = 2 different primary cell cultures from two animals per group). *** *p* < 0.001 by ANOVA followed by Bonferroni post-hoc test.

By qRT PCR, we assessed the expression of the *SOX2* and *OCT4* pluripotency genes in B6.SJL and G93A-SOD1 epSPCs 48 h after administration of FM19G11-loaded NPs. Analysis demonstrated a significant increase of *SOX2* and *OCT4* transcriptional levels in treated as compared to untreated 12-week G93A-SOD1 epSPCs (Figure 2A) and we also observed this increase for 8-week G93A-SOD1 epSPCs, but not 18-week G93A-SOD1 epSPCs (Figure S2). We also observed an increase in *SOX2* and *OCT4* mRNA levels in treated as compared to untreated epSPCs in 12-week B6.SJL epSPCs, although the observed difference failed to reach statistical significance (Figure 2A). In 18-week B6.SJL epSPCs, we observed an increase in *SOX2*, but not *OCT4*, expression in FM19G11-loaded NP treated cells when compared to untreated cells (Figure S2).

In line with the transcriptional data, we also found increased levels of SOX2 and OCT4 protein in 12-week B6.SJL and G93A-SOD1 epSPCs treated with FM19G11-loaded NPs when compared to untreated cells (Figure 2B).

### 3.3. FM19G11-Loaded NPs Increase AKT1 and AKT3 Expression in G93A-SOD1 epSPCs

**Figure 3:**
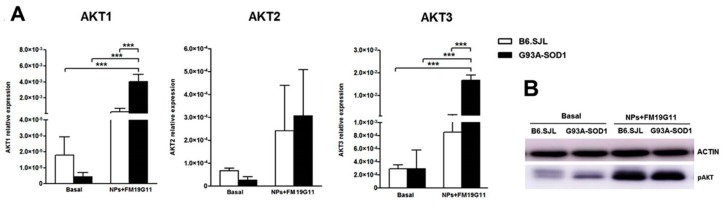
Analysis of AKT1-3 expression following FM19G11-loaded NP treatment (**A**) qRT PCR expression analysis of the *AKT1*, *AKT2*, and *AKT3* genes in 12-week B6.SJL (white bars) and G93A-SOD1 (black bars) epSPCs after 48 h of FM19G11-loaded NP treatment compared to basal conditions. Expression levels reported as mean ± SD of 2^−ΔCT^ values normalized against the endogenous control 18S (*n* = 5 different primary cell cultures from five animals per group). (**B**) Representative Western blot analysis of p-AKT and β-actin (*n* = 2 different primary cell cultures from two animals per group). *** *p* < 0.001 by ANOVA followed by Bonferroni post-hoc test.

We next investigated whether a 48 h incubation of 12-week B6.SJL and G93A-SOD1 epSPCs with FM19G11-loaded NPs affected AKT signaling, a pathway previously shown to be modulated by FM19G11 treatment [14]. By qRT PCR, we demonstrated a significant increase in *AKT1* and *AKT3*, but not *AKT2*, mRNA levels in FM19G11-loaded NP treated G93A-SOD1 epSPCs (Figure 3A) as compared to untreated G93A-SOD1 epSPCs and both treated and untreated B6.SJL epSPCs (Figure 3A). Eight-week G93A-SOD1 epSPCs also demonstrated an increase in *AKT1* and *AKT3* mRNA levels following FM19G11-loaded NP treatment when compared to untreated G93A-SOD1 epSPCs (Figure S3); however, we failed to observe significant differences between treated and untreated week 18 epSPCs (Figure S3).

To confirm the AKT increase at the protein level, we analyzed the expression of the p-AKT protein by Western blot. In line with the mRNA data, we observed higher levels of p-AKT in FM19G11-loaded NP treated epSPCs when compared to untreated in both G93A-DOS1 and B6.SJL (Figure 3B).

### 3.4. FM19G11-Loaded NPs Increase Uncoupling Protein 2 (UCP2) Expression Levels in G93A-SOD1 epSPCs

**Figure 4:**
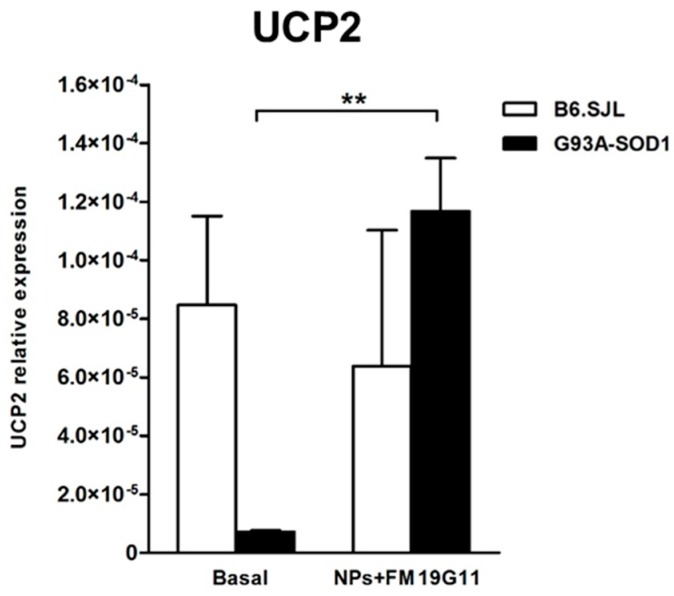
Analysis of *UCP2* Expression Following FM19G11-loaded NP treatment. qRT PCR expression analysis of *UCP2* in 12-week B6.SJL (white bars) and G93A-SOD1 (black bars) epSPCs after 48 h of FM19G11-loaded NP treatment compared to basal conditions. Expression levels reported as mean ± SD of 2^−ΔCT^ values normalized against the endogenous control 18S (*n* = 5 different primary cell cultures from five animals per group). ** *p* < 0.01 by ANOVA followed by Bonferroni post-hoc test.

FM19G11 treatment can induce the expression of mitochondrial genes and mitochondrial activity, thereby triggering an adaptive cellular response which increases ATP production [14]. For this reason, we investigated the expression levels of the *UCP1* and *UCP2* mitochondrial genes in B6.SJL and G93A-SOD1 epSPCs after 48 h of FM19G11-loaded NP treatment. We failed to observe any difference in *UCP1* expression levels between treated and untreated cells in both groups of animals (data not shown); however, we did observe a significant increase of *UCP2* mRNA levels in 12-week (Figure 4; *p* < 0.01) and 8-week (Figure S4; *p* < 0.05), but not 18-week G93A-SOD1 epSPCs (Figure S4) following FM19G11-loaded NP treatment.

### 3.5. FM19G11-Loaded NP Treatment Affects Cell Cycle-Related miRNAs and PTEN Target Gene Expression in G93A-SOD1 epSPCs

**Figure 5:**
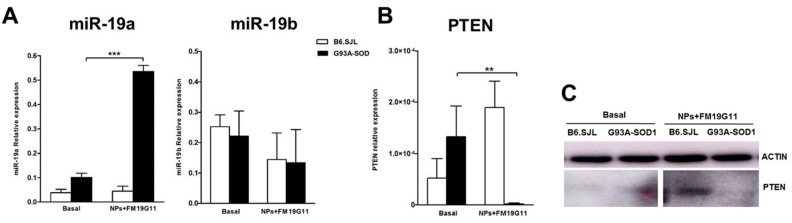
Analysis of cell cycle-related miRNAs and PTEN expression. (**A**) qRT PCR expression analysis of miR-19a and miR-19b in 12-week B6.SJL (white bars) and G93A-SOD1 (black bars) epSPCs after 48 h of FM19G11-loaded NP treatment compared to basal conditions. Expression levels reported as mean ± SD of 2^−ΔCT^ values normalized against the miRNA control U6 (*n* = 5 different primary cell cultures from five animals per group). (**B**) qRT PCR expression analysis of phosphatase and tensin homolog (*PTEN*) target gene in 12-week B6.SJL (white bars) and G93A-SOD1 (black bars) epSPCs after 48 h of FM19G11-loaded NP treatment compared to basal conditions. Expression levels reported as mean ± SD of 2^−ΔCT^ values normalized against the endogenous control 18S (*n* = 5 different primary cell cultures from five animals per group). (**C**) Representative Western blot analysis of PTEN and β-actin (*n* = 2 different primary cell cultures from two animals per group). ** *p* < 0.01, *** *p* < 0.001 by ANOVA followed by Bonferroni post-hoc test.

The observed molecular changes associated with FM19G11-loaded NP treatment suggest the ability of this HIF modulator to increase the proliferative capacity of epSPCs. To further address this idea, we analyzed the expression of the miR-19a and miR19b cell cycle-related microRNAs (miRNAs) in FM19G11-loaded NP treated and untreated epSPCs since studies have indicated the involvement of these miRNAs in the proliferation of G93A-SOD1 epSPCs [11]. Of interest, our analysis established the significant up-regulation of miR-19a, but not of miR-19b, in treated 12-week G93A-SOD1 epSPCs when compared to basal conditions (Figure 5A; *p* < 0.001). For 8-week and 18-week epSPCs in general, we did not observe a significant difference between treated and untreated epSPCs; however, we did observe significantly higher miR-19a levels in treated as compared to untreated 18-week B6.SJL epSPCs (Figure S5).

We next explored the expression levels of phosphatase and tensin homolog (*PTEN*), a gene target of miR-19a involved in the proliferation and self-renewal of epSPCs [11]. Notably, in 12-week G93A-SOD1 epSPCs, *PTEN* expression displayed an opposite trend compared to miR-19a. Indeed, *PTEN* transcript levels were significantly down-regulated in these epSPCs after treatment with FM19G11-loaded NPs (Figure 5B; *p* < 0.01). In 8- and 18-week epSPCs, we failed to observe a significant difference in *PTEN* expression between treated and untreated cells (Figure S5). In line with our mRNA data, Western blot analysis established a decrease in PTEN protein expression in treated 12-week G93A-SOD1 epSPCs when compared to basal conditions (Figure 5C). Of note, these results correlate well with those found for AKT expression, as PTEN negatively regulates AKT [23]. However, we did not find a decrease in PTEN levels in 12- (Figure 5B,C) or 18-week (Figure S5) B6.SJL epSPCs. This differential response could derive from the differential expression of miR-9, a known regulator of PTEN expression, previously reported by our group to be overexpressed in G93A-SOD1 epSPCs in comparison to B6.SJL epSPCs [11].

## 4. Discussion

We currently lack effective therapies that can prevent or reduce motor neuron degeneration in ALS [24]. Previous data from our own and other groups have demonstrated that motor neuronal degeneration in G93A-SOD1 mice activates the proliferation of epSPCs, although these cells still cannot support motor neuron survival [9,10,11]. Thus, the possibility of further increasing epSPC proliferation, differentiation capacity, and cell viability could represent a future therapeutic strategy to slow down neurodegeneration and disease progression. Recent studies have shown how FM19G11, an hypoxia-inducible factor inhibitor, impacts the self-renewal and proliferation of epSPCs [14].

The present study aimed to investigate the effect of FM19G11 on the self-renewal and proliferation of epSPCs derived from G93A-SOD1 mice at different disease stages. We chose to test the potential therapeutic effect of FM19G11 at disease onset (12 weeks) to improve the translational applicability of our findings. We decided to extend our investigation to the pre-symptomatic stage of the disease (8 weeks) since pathological alterations can be detected in G93A-SOD1 mice at this time [8], while studies have shown that epSPCs attempt to proliferate in response to neuronal damage in ALS [11]. Additionally, we included a time point representative of late-stage disease (18 weeks) to evaluate whether highly damaged motor neurons may also be affected by FM19G11 treatment. As FM19G11 is hydrophobic, making treatment with the free drug difficult, we loaded FM19G11 in the ligand shell of gold NPs and evaluated efficacy when using this formulation. We obtained data indicative of a significant increase in the number of G93A-SOD1 and B6.SJL epSPCs following treatment with FM19G11-loaded NPs compared to cells treated with “free” FM19G11 and untreated controls. These results are in accordance with previous data showing increased proliferation of epSPCs by FM19G11 in a rat model of spinal cord damage [14]. Moreover, the present data demonstrated that FM19G11 is more effective when delivered as part of an NP formulation, further enhancing the proliferative and self-renewal capacity of epSPCs derived from both G93A-SOD1 and B6.SJL mice. We hypothesize that NP-mediated FM19G11 delivery allows for a more concentrated dose delivered to cells. Of note, we failed to observe drug leakage from NPs in solution (data not shown). NPs alone had no effect on epSPC proliferation, not beneficial in terms of increased cell proliferation, nor adverse in terms of nanotoxicity. There are numerous clinical applications involving NPs for the delivery of specific molecules into damaged regions for the treatment of neurodegenerative diseases, including ALS [25,26,27,28,29]. Previous studies highlighted the ability of NPs to release hydralazine in neurons to inhibit the mitochondrial alterations identified in motor neurons of ALS patients [30,31].

We confirmed the impact of FM19G11-loaded NPs on the proliferation of epSPCs by gene expression analysis of *TERT*, a ribonucleoprotein polymerase essential for the replication of chromosome ends [13]. We observed increased mRNA levels of *TERT* in G93A-SOD1 epSPCs after treatment, while Rodríguez-Jiménez and colleagues [14] found similar results in epSPC cultures isolated from a rat model of spinal cord injury. Overall, this highlights the positive, relevant effect of FM19G11 on epSPC proliferation and self-renewal. Furthermore, our study reports the phosphorylation and potential activation of AKT, involved in the PI3K/AKT cell survival signal transduction pathways, in epSPCs following FM19G11 treatment. While AKT enzymes are poorly expressed or inactivated in ALS pathogenesis [32], the constitutive expression of AKT3 in motor neurons from G93A-SOD1 mice provides a neuroprotective effect with an increase of motor neuron survival when compared to cells expressing low levels of the enzyme in vitro and in vivo [33]. An increase in cell survival and neuroprotective processes in G93A-SOD1 motor neurons was also associated with AKT activation following induction of hypoxia in vivo [34] and the up-regulation of anti-apoptotic factors in vitro [32]. In addition, Li and colleagues [35] demonstrated the activation of the PI3K/AKT signal transduction pathways associated with reduced cell death in G93A-SOD1 motor neurons, which were characterized by high toxicity caused by the presence of reactive oxygen species (ROS) and treated with vascular endothelial growth factor. In line with previous studies, our analyses demonstrated the increased expression of *AKT1* and *AKT3* mRNAs in G93A-SOD1 and B6.SJL epSPCs treated with FM19G11-loaded NPs when compared to untreated cells. In a similar manner to epSPC number and TERT expression, *AKT1* and *AKT3* levels were particularly elevated in FM19G11-loaded NP treated 8- and 12-week G93A-SOD1 epSPCs, corresponding to the onset of the disease. In the murine model, the onset of disease correlates with this first evident clinical signs of disease [7] and, so, the stage at which cell function and pathways become altered, but perhaps still susceptible to modulation or normalization. This could explain the lack of effect of FM19G11-loaded NPs in cells from ALS mice at 18 weeks of age, representing the late stages of the disease. In humans, the 8–12 week phase can be compared to the stage in which the first observable clinical manifestations are present when patients generally undergo therapy [35]. Thus, the elevated activity of FM19G11 at the onset of disease in ALS mice suggests the possible application of this molecule in therapeutic approaches aimed at delaying disease progression in newly diagnosed ALS patients.

In the cells representative of the advanced stages of disease (18 weeks), we failed to observe a neuroprotective effect of FM19G11 on the PI3K/AKT activation cell survival pathway. At advanced stages of disease, additional mechanisms may be required to rescue dying neurons. Indeed, the mitochondrial damage and pathological protein aggregates caused by mutations in the SOD1 gene in epSPCs can hamper the neuroprotective role induced by FM19G11.

Several studies have demonstrated that the UCP decoupling proteins exert a neuroprotective effect in ALS. Indeed, Andrews and colleagues discovered a correlation between ROS reduction and UCP2 protein activation in the mitochondria of neuronal cells [36]. UCP2 activation allowed the induction of mitochondrial biogenesis, while the initial induction of mitochondrial decoupling and decrease in ATP levels can act as triggers for the increase of the number of mitochondria and ATP in neuronal tissues [36] and in epSPCs isolated from a rat model of spinal cord injury [14]. Notably, our analysis unequivocally demonstrated an increase in *UCP2* expression levels in G93A-SOD1 epSPCs treated with FM19G11-loaded NPs compared to untreated cells, and again, we observed this increase specifically in cells isolated from mice at the onset of disease (8 and 12 weeks). These results suggest that FM19G11 regulates mitochondrial activity and protects G93A-SOD1 epSPCs by modulating the expression of the UCP2 uncoupling protein.

Our findings also discovered an increase in the expression of the SOX2 and OCT4 pluripotency and self-renewal factors [11,13,37] in 8- and 12-week G93A-SOD1 epSPCs treated with FM19G11-loaded NPs compared to untreated cells. These results highlight the crucial role of FM19G11 in promoting epSPC self-renewal and maintaining multipotentiality. We also observed that FM19G11 treatment increased the expression levels of miR-19a in 12-week G93A-SOD1 epSPCs, but decreased the expression of the miR-19a gene target *PTEN*. Our recent data highlighted the implication of this miRNA in the cell cycle and self-renewal of G93A-SOD1 epSPCs [11], thus supporting the ability of FM19G11 to regulate RNA levels through a significant up-regulation of miR-19a and a down-regulation of its target gene *PTEN*, accompanied by the induction of the PI3K/AKT cell survival pathway. Molecular mechanisms which correlate PTEN inhibition with PI3K/AKT activation were demonstrated by Kirby and colleagues in G93A-SOD1 motor neurons [32], indicating that the proteins involved in these signal transduction pathways could represent therapeutic targets for motor neuron diseases. The previously observed increase in miR-19a expression levels in epSPCs derived from G93A-SOD1 mice was encountered in the symptomatic phase of the disease (week 18) [11]. Here, FM19G11 delivered by NPs induced miR-19a up-regulation in G93A-SOD1 epSPCs at earlier stages of the disease (weeks 8 and 12), when epSPCs are quiescent in ALS mice [11]. This suggests that treatment with FM19G11-loaded NPs could represent an efficient method for the early modulation of epSPC proliferation, with the hope of reducing the neurodegeneration typical of ALS.

We are aware of the need to validate our in vitro findings in an in vivo model; however, although the G93A-SOD1 mouse resembles the human disease, it represents an animal model of fALS, and in particular, fALS involving a mutation in the SOD1 gene, and may not model the sporadic form of ALS. Furthermore, confounding biological variables may be present in the animal model that might influence the results. However, we remain confident that our observation has relevance for the human disease, as we have recently observed the beneficial effect of FM19G11 on human cell lines (unpublished results) and because of the positive effect of the treatment on cells representative of disease onset.

## 5. Conclusions

Our findings highlight the beneficial effects of FM19G11 on neuroprotection, self-renewal, and proliferation of epSPCs isolated from the spinal cord of G93A-SOD1 mice when delivered by NP. We believe that this effect derives from the ability of FM19G11 to activate PI3K/AKT and UCP2 signal transduction pathway that influences stemness, self-renewal, and proliferation through the induction of the *SOX2*, *OCT4*, and *TERT* genes and miR-19a. This evidence may open new avenues for the future development of novel therapeutic approaches based on FM19G11-loaded NPs to slow neurodegeneration and disease progression in ALS patients.

## Supplementary Materials

Supplementary materials are available online https://www.mdpi.com/2073-4409/8/3/279/s1. Figure S1. Effect of FM19G11 treatment on percentage of ependymal stem progenitor cells (epSPCs) isolated from G93A-SOD1 and control mice at weeks 8 and 18, and on the expression of the mRNA reverse transcriptase (TERT). Figure S2. Expression levels of SOX2 and OCT4 pluripotency markers in epSPCs isolated from G93A-SOD1 and control mice at weeks 8 and 18. Figure S3. Expression levels of AKT1, AKT2 and AKT3 genes in epSPCs isolated from G93A-SOD1 and control mice at weeks 8 and 18. Figure S4. Expression levels of UCP2 gene in epSPCs isolated from G93A-SOD1 and control mice at weeks 8 and 18. Figure S5. Expression levels of miR-19a and -19-b and their target gene PTEN in epSPCs isolated from G93A-SOD1 and control mice at weeks 8 and 18.
