# Supplementary material for: FM19G11-Loaded Gold Nanoparticles Enhance the Proliferation and Self-Renewal of Ependymal Stem Progenitor Cells Derived from ALS Mice
Source: Cells. 2019 Mar 23;8(3):279. doi: 10.3390/cells8030279 (PMC6468696; doi:10.3390/cells8030279)
Supplement: Supplementary file 1 [file cells-08-00279-s001.pdf]

Supplementary Figures

Figure S1. Effect of FM19G11 treatment on percentage of ependymal stem progenitor cells (epSPCs) isolated from G93A-SOD1 and control mice at weeks 8 and 18, and on the expression of the mRNA reverse transcriptase (TERT).

Figure S2. Expression levels of SOX2 and OCT4 pluripotency markers in epSPCs isolated from G93A-SOD1 and control mice at weeks 8 and 18.

Figure S3. Expression levels of AKT1, AKT2 and AKT3 genes in epSPCs isolated from G93A-SOD1 and control mice at weeks 8 and 18.

Figure S4. Expression levels of UCP2 gene in epSPCs isolated from G93A-SOD1 and control mice at weeks 8 and 18.

Figure S5. Expression levels of miR-19a and -19-b and their target gene PTEN in epSPCs isolated from G93A-SOD1 and control mice at weeks 8 and 18.

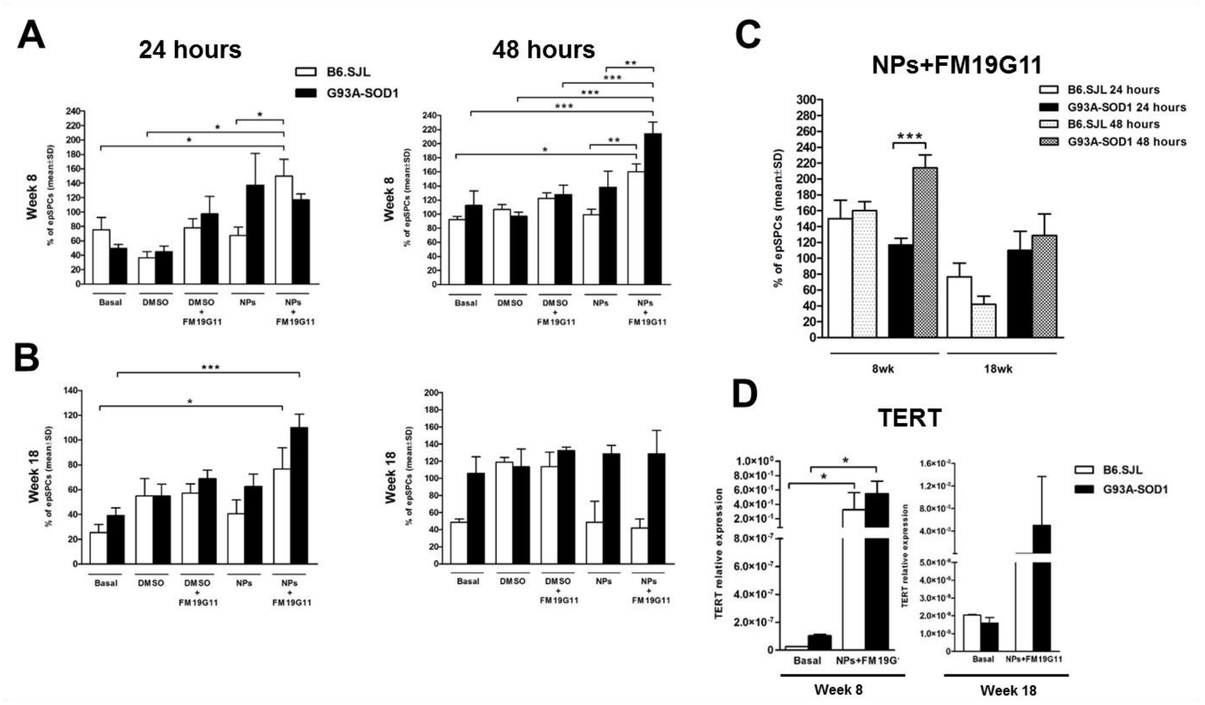

Figure S1. Effect of FM19G11 treatment on percentage of ependymal stem progenitor cells (epSPCs) isolated from G93A-SOD1 and control mice at weeks 8 and 18, and on the expression of the mRNA reverse transcriptase (*TERT*). Percentage of epSPCs from B6.SJL (white bars) and G93A-SOD1 mice (black bars) at weeks 8 (**A**) and 18 (**B**) after 24 and 48 hours under the following growth conditions: 1) basal condition (only medium); 2) treatment with 500 nM of FM19G11 in DMSO (Sigma), and separately the corresponding amount of vehicle as control; 3) treatment with 500 nM FM19G11 bound to 0.01 mg/mL NPs, and separately the corresponding amount of vehicle. Data are presented as mean ± SD of percentage of epSPCs (n = 10 cell lines for each group). (**C**) Percentage of epSPCs isolated from B6.SJL and G93A-SOD1 mice at 8 and 18 weeks after 24 (white and black bars) and 48 (white and black dot patterns) hours of treatment with FM19G11 bound to nanoparticles. Data are presented as mean ± SD of percentage of epSPCs (n = 10 different cell lines from 10 animals per group for each time point). (**D**) Real-time PCR expression analysis of *TERT* gene in B6.SJL (white bars) and G93A-SOD1 (black bars) epSPCs at weeks 8 and 18 after 48 hours of treatment with FM19G11-loaded nanoparticles and at basal condition. Expression levels are reported as mean ± SD of $2^{-\Delta CT}$ values normalized against the endogenous control 18S (n = 5 different primary cell cultures from 5 animals per group for each time point). * $p < 0.05$, ** $p < 0.01$, *** $p < 0.001$ One way analysis of variance (ANOVA), followed by Bonferroni post-hoc test.

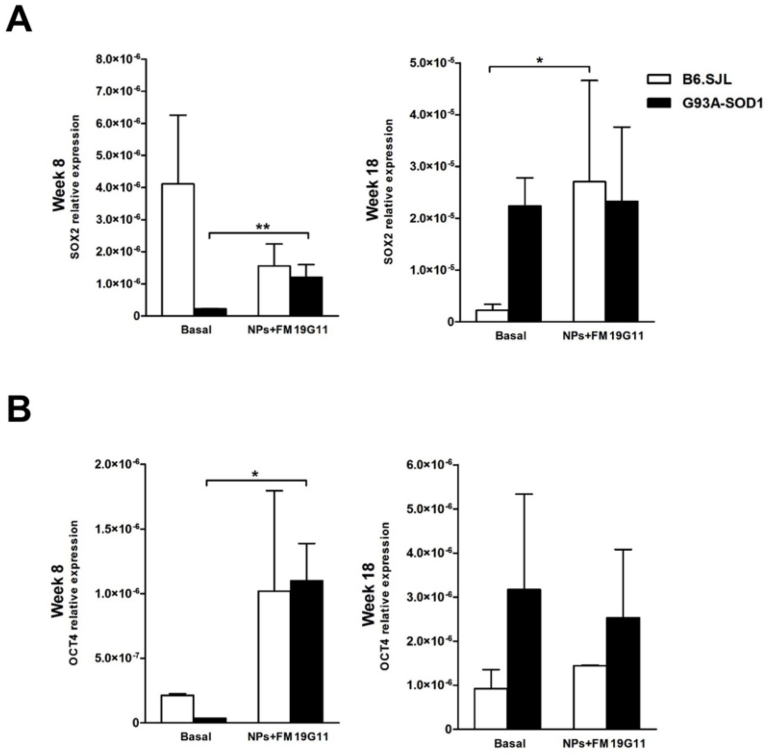

Figure S2. Expression levels of *SOX2* and *OCT4* pluripotency markers in epSPCs isolated from G93A-SOD1 and control mice at weeks 8 and 18. Real-time PCR expression analysis of *SOX2* (**A**) and *OCT4* (**B**) genes in B6.SJL (white bars) and G93A-SOD1 (black bars) epSPCs from animals at weeks 8 and 18 of age after 48 hours of FM19G11-loaded nanoparticle treatment and at basal condition. Expression levels are reported as mean ± SD of $2^{-\Delta CT}$ values normalized against the endogenous control 18S (n = 5 different primary cell cultures from 5 animals per group for each time point). * $p < 0.05$, ** $p < 0.01$, One way analysis of variance (ANOVA), followed by Bonferroni post-hoc test.

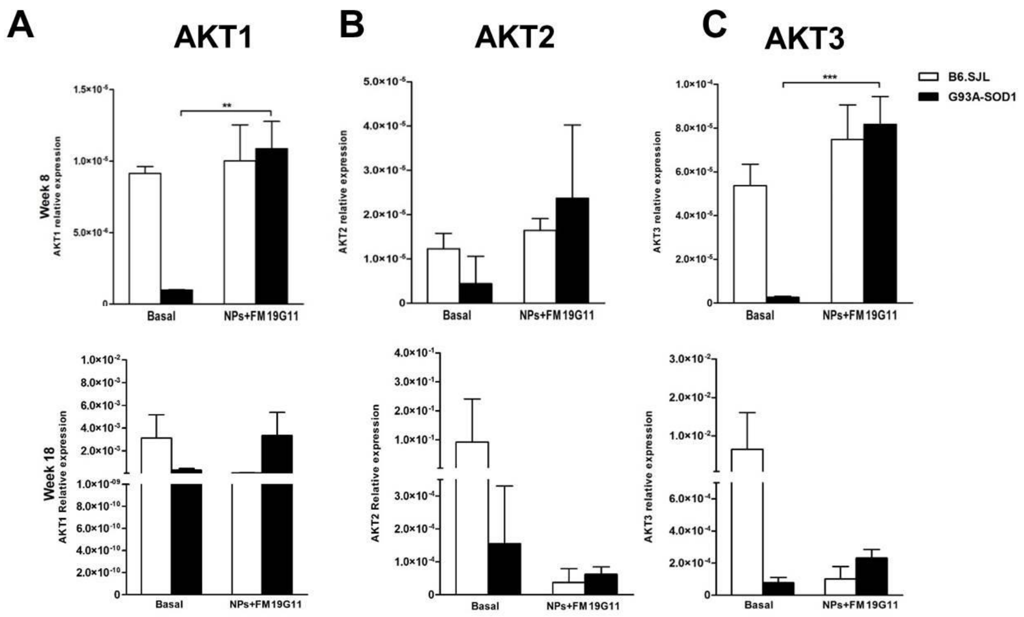

Figure S3. Expression levels of *AKT1*, *AKT2* and *AKT3* genes in epSPCs isolated from G93A-SOD1 and control mice at weeks 8 and 18. Real-time PCR expression analysis of *AKT1* (**A**), *AKT2* (**B**) and *AKT3* (**C**) genes in B6.SJL (white bars) and G93A-SOD1 (black bars) epSPCs from animals at weeks 8 and 18 after 48 hours of FM19G11-loaded nanoparticle treatment and at basal condition. Expression levels are reported as mean ± SD of $2^{-\Delta CT}$ values normalized against the endogenous control 18S (n = 5 different primary cell cultures from 5 animals per group for each time point). ** $p < 0.01$, *** $p < 0.001$, One way analysis of variance (ANOVA), followed by Bonferroni post-hoc test.

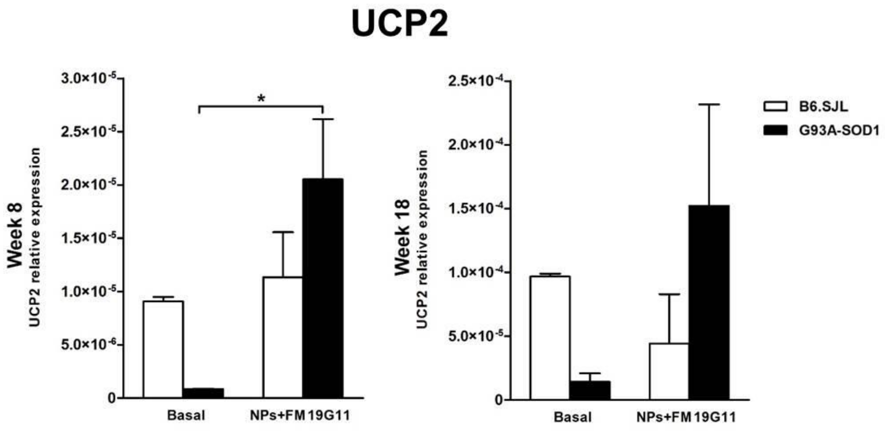

Figure S4. Expression levels of *UCP2* gene in epSPCs isolated from G93A-SOD1 and control mice at weeks 8 and 18. Real-time PCR expression analysis of *UCP2* in B6.SJL (white bars) and G93A-SOD1 (black bars) epSPCs from animals at weeks 8 and 18 after 48 hours of FM19G11-loaded nanoparticle treatment and at basal condition. Expression levels are reported as mean ± SD of $2^{-\Delta CT}$ values normalized against the endogenous control 18S (n = 5 different primary cell cultures from 5 animals per group for each time point). * $p < 0.05$, One way analysis of variance (ANOVA), followed by Bonferroni post-hoc test.

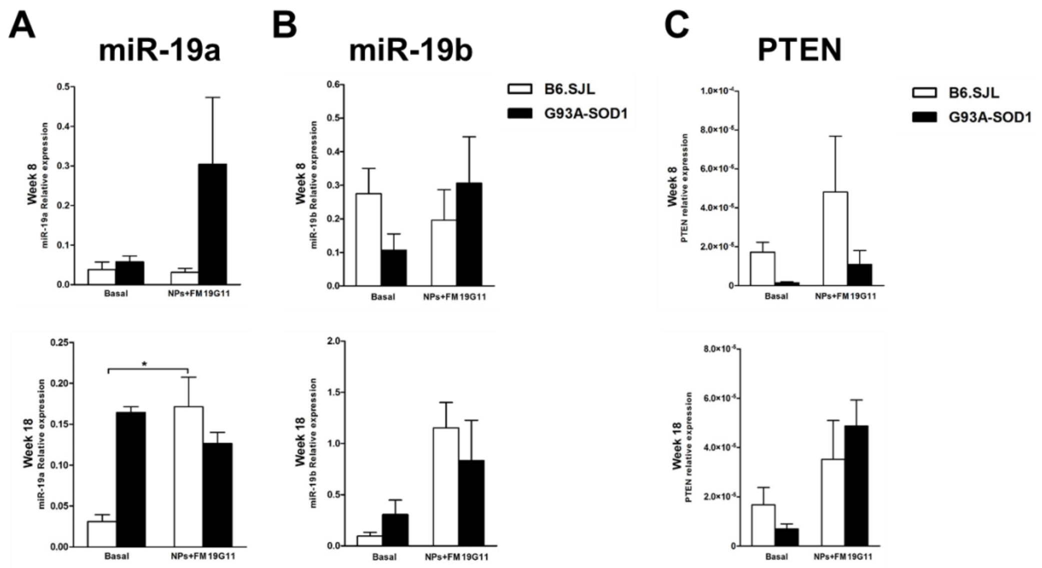

Figure S5. Expression levels of miR-19a and -19-b and their target gene *PTEN* in epSPCs isolated from G93A-SOD1 and control mice at weeks 8 and 18. Real-time PCR expression analysis of miR-19a (**A**) and miR-19b (**B**) in B6.SJL (white bars) and G93A-SOD1 (black bars) epSPCs from mice at weeks 8 and 18 after 48 hours of FM19G11-loaded nanoparticle treatment and at basal condition. Expression levels are reported as mean ± SD of $2^{-\Delta CT}$ values normalized against the miRNA control U6 (n = 5 different primary cell cultures from 5 animals per group for each time point). (**C**) Real-time PCR expression analysis of *PTEN* target gene in B6.SJL (white bars) and G93A-SOD1 (black bars) epSPCs from mice at weeks 8 and 18 after 48 hours of FM19G11-loaded nanoparticle treatment and at basal condition. Expression levels are reported as mean ± SD of $2^{-\Delta CT}$ values normalized against the endogenous control 18S (n = 5 different primary cell cultures from 5 animals per group for each time point). * $p < 0.05$, One way analysis of variance (ANOVA), followed by Bonferroni post-hoc test.
